# Detection of nephrotoxic drugs and dose adjustment in patients with low glomerular filtration rate in community pharmacy: A multicenter study in Spanish community pharmacies

**DOI:** 10.1371/journal.pone.0333345

**Published:** 2025-10-14

**Authors:** Gema Escribá-Martí, Iker Cámara-Ramos, María Teresa Climent-Catalá, Verónica Escudero-Quesada, Luis Salar-Ibáñez

**Affiliations:** 1 Community Pharmacist in Segart, Valencia, Spain; 2 Community Pharmacist in Bilbao, Bilbao, Spain; 3 PharmD, President of the SEFAC Delegation in the Valencian Community, Community Pharmacist in Valencia, Spain; 4 Nephrologist, Nephrology Service, Dr. Peset University Hospital, Valencia, Spain; 5 PharmD, Cardenal Herrera University–CEU, Community Pharmacist in Valencia, Valencia, Spain; University of Toronto Temerty Faculty of Medicine, CANADA

## Abstract

Chronic kidney disease (CKD) represents a significant global health burden, with its prevalence markedly increasing in the elderly. This multicenter, non-controlled, prospective study was conducted in 16 Spanish community pharmacies to assess the feasibility of identifying potentially inappropriate prescriptions (PIPs) in patients with reduced estimated glomerular filtration rate (eGFR). Patients over 60 years old with a body mass index between 19 and 35 kg/m^2^, who were taking at least one nephrotoxic medication or a drug requiring dose adjustment based on renal function, were recruited. A point-of-care (POC) test for creatinine using the StatSensor Xpress® analyzer was performed for those lacking an eGFR measurement in the previous three months, with eGFR calculated via the CKD-EPI formula. Of the 670 patients recruited (mean age 72.5 ± 9.3 years; 49.9% female), 455 had an eGFR > 60 ml/min/1.73 m^2^, while 215 had values below this threshold. The medication of these latter patients was reviewed, and in 90 of them (41.9%) a potentially inappropriate prescription was identified, leading to their referral to their primary care physician. Of the total sample, 13.4% of the patients had at least one PIP. Changes were requested for 9.0% of the medications, and these were implemented in 3.4%. The proportion of PIPs increased with CKD severity, with higher intervention rates observed in advanced stages. These findings highlight the potential of community pharmacies to contribute to early CKD risk detection and medication optimization, emphasizing the need for enhanced direct communication channels between pharmacists and physicians to improve patient outcomes.

## Introduction

Chronic kidney disease (CKD) is characterized by the persistent elevation of urinary albumin excretion (≥30 mg/g [≥3 mg/mmol]) or a sustained reduction in the estimated glomerular filtration rate (eGFR < 60 ml/min/1.73 m^2^), or both, for a period exceeding three months [1]. CKD represents a significant global health concern. According to a World Health Organization publication, it is regarded as “the most neglected chronic disease” [2], with a worldwide prevalence estimated at 13.4% (95% CI: 11.7–15.1).

The KDIGO (Kidney Disease: Improving Global Outcomes) guidelines [3] classify Chronic Kidney Disease (CKD) into seven stages based on glomerular filtration rate (GFR): G1, GFR > 90 mL/min/1.73 m^2^; G2, GFR 60–89 mL/min/1.73 m^2^; G3a, GFR 45–59 mL/min/1.73 m^2^; G3b, GFR 30–44 mL/min/1.73 m^2^; G4, GFR 15–29 mL/min/1.73 m^2^; and G5, GFR < 15 mL/min/1.73 m^2^

The most common stage is 3a which has a prevalence of 7.6% (95% CI: 6.4–8.9) [4]. In Spain, data from the ENRICA study (Estudio de Nutrición y Riesgo Cardiovascular en España) reveal that 15.1% (95% CI: 14.3–16.0) of the population is affected by CKD, with stage 3a also being the most prevalent (10.0% (95% CI: 9.3–10.8]) [5]. Furthermore, CKD prevalence increases markedly with age, reaching 37.3% in individuals over 65 years of age [5,6]. Over time, renal function progressively declines, with the mean annual decrease in GFR estimated at 0.8 ml/min/1.73 m^2^ in women and 1.4 ml/min/1.73 m^2^ in men [7].

In addition to age, diabetes and hypertension represent the primary risk factors for the development of CKD [8]. In patients younger than 40 years, the multivariable-adjusted hazard ratio (HR) for end-stage renal disease was 2.18 for systolic blood pressure (SBP) ≥160 mm Hg, and 4.52 for diastolic blood pressure (DBP) ≥110 mm Hg. In contrast, among patients older than 70 years, the HR was 1.84 for SBP ≥ 160 mm Hg and 2.34 for DBP ≥ 110 mm Hg [9]. The prevalence of CKD in patients with type 2 diabetes mellitus was 31.4%, with an under-reporting rate of 47%. Metformin was the principal therapeutic agent for diabetes management in both CKD and non-CKD patients, although its use is contraindicated at certain stages of CKD [10]. Additional risk factors include cardiovascular disease, dyslipidemia, obesity, smoking, hypoalbuminemia, and hyperuricemia [1,11], many of which are also prevalent in the elderly. Approximately 1% of patients with chronic kidney disease (CKD) eventually require renal replacement therapy, either through dialysis, which significantly reduces both quality and life expectancy [5], or through kidney transplantation, which achieves much higher levels of life expectancy and quality of life.

According to the National Transplant Organization, 4047 kidney transplants were performed in Spain in 2024 [12].

Early detection of the disease is crucial for preventing mortality and morbidity; however, this is challenging because it is typically asymptomatic during its initial stages. Primary care represents the optimal setting for identifying these asymptomatic individuals, particularly among patients with diabetes, hypertension, cardiovascular disease, or a family history of CKD [13]. Treatment strategies focus on renal protection by managing complications and mitigating risk factors, especially hypertension and diabetes, that may worsen the disease [14]. Moreover, it is important to identify and manage nephrotoxic drugs in patients with stage 3 CKD and to discontinue them in stages 4–5 [13–15]. This includes adjusting drug dosages according to eGFR to avoid further nephrotoxicity or potential overdosing due to decreased renal clearance [16–18]. These two aspects are collectively referred to as Potentially Inappropriate Prescribing (PIP). Studies have shown that up to 52% of patients who require such adjustments are identified [17].

### Role of community pharmacy

Due to its availability and accessibility, the community pharmacy may be considered an extension of primary care services. Its healthcare capabilities are expanding in Spain and other countries, notably the Netherlands and Canada. These capabilities include services related to CKD, such as the screening and detection of asymptomatic patients [18,19], medication advice and reconciliation at hospital discharge [20], and subsequent follow-up to identify medication-related complications, including non-adherence [21]. Moreover, a 2019 study conducted in the Netherlands utilized point-of-care testing for kidney function (GFR) in community pharmacies to prevent hospitalizations related to antibiotic adverse events [22]. The study reported an annual saving of €86 per patient, primarily due to the avoidance of hospitalizations.

Community pharmacies are exceptionally well-positioned to conduct screening, as has been consistently demonstrated in Spain and other countries [23–26]. With approximately two million individuals visiting Spanish pharmacies daily, many of whom do not regularly consult a physician, pharmacies serve as an ideal venue for detecting occult diseases, including CKD. Specifically, regarding CKD, community pharmacies can provide substantial benefits to both the Public Health System and the population by facilitating early screening and detection, as well as by reviewing medications to identify inappropriate use of nephrotoxic drugs and to recommend dose adjustments for renally cleared medications [26]. In Spain, the lack of direct communication between community pharmacies and primary care is a well-recognized issue. Consequently, pharmacists rely solely on information from the electronic prescription system and on patient-provided data.

### Objectives

- Identify and alert physicians regarding the use of nephrotoxic drugs in these patients.
- Identify and notify physicians about excessive dosing based on patients’ GFR.

## Materials and methods

This study is a prospective, non-controlled, follow-up, experimental, analytical, multicenter investigation. Sixteen community pharmacies from the Valencian Community, the Basque Country, Galicia, and the Principado de Asturias participated. To ensure homogeneity in the intervention and data recording procedures, all pharmacies received a four-hour classroom training session during which the study protocol and the use of the POC meter for obtaining the glomerular filtration rate were thoroughly explained. Subsequently, the pharmacists were afforded the opportunity to resolve any questions regarding the study procedures or specific patient cases.

The recruitment period lasted from March 13, 2020, to June 20, 2022.

Patient inclusion criteria

Patients presenting to the pharmacy for any reason were eligible for inclusion if they met the following criteria:

- Age over 60 years
- Body mass index between 19 and 35 kg/m^2^
- Use of at least one nephrotoxic medication or a medication requiring dose adjustment based on eGFR

All participants were required to provide written informed consent.

Exclusion criteria

Patients were excluded if they met any of the following conditions:

- Adherence to special diets (e.g., strict vegetarianism, creatinine or creatine supplementation) or presence of malnutrition
- Alterations in muscle mass due to amputations, significant muscle loss, neuromuscular diseases, or paralysis
- Severe liver disease, generalized edema, or ascites
- Acute kidney failure
- Current dialysis treatment or being on the waiting list for kidney transplantation

### Procedure

Patients meeting the inclusion criteria were asked whether they had an eGFR measurement within the previous three months. If no recent measurement was available, they were offered a point-of-care test for capillary blood creatinine using the StatSensor Xpress® analyzer (Nova Biomedical, Waltham, MA), with eGFR subsequently calculated via the CKD-EPI formula. The validity of this analyzer was confirmed for this study [27].

For patients with a GFR below 60 ml/min/1.73 m^2^, their medication regimens were evaluated for nephrotoxic agents or drugs requiring dose adjustments based on eGFR. This assessment was performed using the ChekTheMeds® web application [28], the Consensus Guide for the Use of Drugs in Kidney Failure [29], and the technical data sheets of the involved medications.

Potentially Inappropriate Prescribing (PIP) was defined as the prescription of either a contraindicated drug or a drug administered at an inappropriately high dose relative to the patient’s eGFR as determined by the CKD-EPI equation [16]. When such medications were identified, a report was generated for the Primary Care Physician and provided to the patient in an open envelope.

Subsequently, data were collected on any modifications made by the Primary Care Physician in response to the intervention.

Based on the medication review conducted by the pharmacist, patients are classified into three groups:

- Those not requiring a medication review
- Those requiring a medication review
- Those referred to a physician

All data were recorded in a Microsoft Access database specifically designed for the study. Although personal patient data were retained by the pharmacies for follow-up purposes, anonymized data were sent to the investigator to ensure patient confidentiality.

### Ethical considerations

This study was conducted in compliance with the Good Clinical Practice guidelines of the International Conference on Harmonization (ICH E6) [30] for studies of this nature. Participant autonomy was respected in every instance, in accordance with the ethical principles of the Declaration of Helsinki and the Organic Law 3/2018 of December 5, on the Protection of Personal Data and Guarantee of Digital Rights.

The collaborating pharmacist provided detailed verbal and written information to the patients regarding the study’s purpose and characteristics. It was emphasized that participation was entirely voluntary and that participants could withdraw at any time. Written informed consent was obtained from all patients, with assurances given regarding the absolute confidentiality of their data.

The study protocol was approved by the Medical Research Ethics Committee of the Dr. Peset University Hospital (CEIm:66/19).

### Statistical analysis

Descriptive statistics were calculated using both mean and median values. For group comparisons, the chi-square test was employed for qualitative variables, while Student’s t-test, adjusted for equal or unequal variances as appropriate, was used for quantitative variables. Differences were deemed statistically significant when p < 0.05.

## Results

Between March 13, 2020, and June 20, 2022, 16 community pharmacies across four Autonomous Communities enrolled 670 patients, mean age of 72.5 years (SD = 9.3), 49.9% female. Of these patients, 455 exhibited an eGFR above 60 ml/min/1.73 m^2^ and therefore did not require further evaluation.

Table 1 illustrates the distribution of CKD stages by sex, along with the corresponding age distribution. While no significant differences were observed between sexes (p = 0.261), age differences were statistically significant (p < 0.001).

**Table 1:** Distribution by sex and age in the different stages of CKD.

CKD stage (GFR)	Male n (%)	Female n (%)	<70 n (%)	70-79 n (%)	80-89 n (%)	≥90 n (%)
1 (>90)	57 (17,0)	50 (15,0)	62 (26,4)	39 (13,3)	6 (3,7)	0 (0,0)
2 (>60 AND <90)	171 (50,9)	177 (53,0)	122 (51,9)	164 (56,0)	58 (35,6)	4 (44,4)
3a (>45 AND <60)	62 (18,5)	64 (19,2)	36 (15,3)	57 (19,5)	58 (35,6)	2 (22,2)
3b (>30 AND <45)	35 (10,4)	39 (11,7)	12 (5,1)	31 (10,6)	31 (19,0)	3 (33,3)
4 (>15 AND < 30)	11 (3,3)	4 (1,2)	3 (1,3)	2 (0,7)	10 (6,1))	0 (0,0)
5 (> 15)	0 (0,0)	0 (0,0)	0 (0,0)	0 (0,0)	0 (0,0)	0 (0,0)
Total	336 (100)	334 (100)	235	293	133	9

Table 2 presents the mean GFR along with its standard deviation stratified by age group.

**Table 2:** Mean GFR and standard deviation by age group.

Age	n	Average	SD
<70	235	75,6	18,7
70-79	293	69,3	18,7
80-89	133	58,4	20,0
≥90	9	55,9	18,1

Among the 215 patients with an eGFR below 60 ml/min/1.73 m^2^ who were further evaluated, 125 were receiving appropriate treatment for their renal condition, while 90 patients were identified as having at least one potentially inappropriate prescription (PIP) and were referred to their primary care physician.

Within this subgroup of 90 patients, 11 were using contraindicated medications—prompting a request for withdrawal; 58 were prescribed medications at doses exceeding the recommended levels for their eGFR, leading to a request for dose adjustment; and 21 patients required both medication withdrawal and dose adjustment.

**Fig 1:**
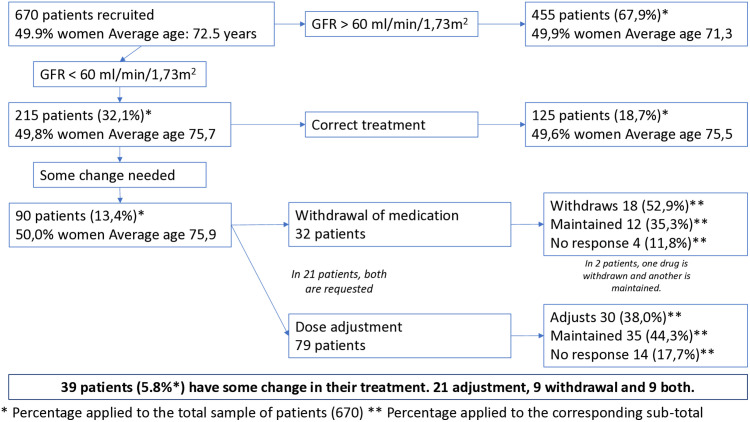
Illustrates the distribution of patients throughout the study procedure. *Percentage applied to the total sample of patients (670). ** Percentage applied to the corresponding sub-total.

Details can be seen in Fig 1.

A total of 670 patients were enrolled in the study, collectively taking 4514 medications. Among the 455 patients with an eGFR > 60 ml/min/1.73 m^2^, 2843 drugs were prescribed, corresponding to an average of 6.2 (SD 3.2) drugs per patient. In contrast, the 215 patients with an eGFR < 60 ml/min/1.73 m^2^ were taking 1670 drugs, with an average of 7.8 (SD 4.1) drugs per patient (p < 0.0001).

Within the group of patients with an eGFR < 60 ml/min/1.73 m^2^, those on an appropriate treatment regimen (n = 125) were prescribed 848 medications, yielding an average of 6.8 (SD 3.9) drugs per patient. Conversely, the 90 patients with at least one potentially inappropriate prescription (PIP) were taking 823 medications, averaging 9.1 (SD 3.9) drugs per patient (p < 0.0001).

The observed difference in the number of medications per patient appears to be more closely related to treatment adequacy than to renal function per se. Specifically, no significant differences were detected in the number of drugs prescribed to patients with an eGFR > 60 ml/min/1.73 m^2^ compared to those with an eGFR < 60 ml/min/1.73 m^2^ who were receiving appropriate treatment (p = 0.1484) (See Table 3).

**Table 3:** Number of medications in the different patient groups.

		Patients	n	Average	SD	p	
GFR > 60		455	2843	6,2	3,2	0,1484	
GFR < 60	Correct treatment	125	848	6,8	3,9		<0,0001
	Some change needed	90	823	9,1	3,9		

A total of 150 medication changes were proposed for 90 patients. Of these proposals, 57 modifications were implemented, corresponding to 38% of the proposed changes and representing 3.4% of the 1,670 medications evaluated.

**Fig 2:**
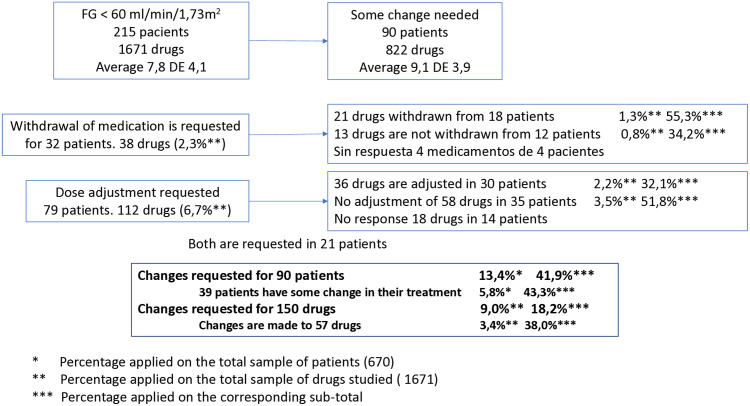
Distribution of medicines throughout the procedure. *Percentage applied on the total sample of patients (670). **Percentage applied on the total sample of drugs studied (1671). ***Percentage applied on the corresponding sub-total.

Further details are provided in Fig 2.

The proportion of patients with potentially inappropriate prescriptions (PIP), as well as the number of dose-adjusted and contraindicated medications, increased with advancing CKD stage (p < 0.0001, p = 0.0127, and p < 0.0001, respectively). Although the proportion of drugs that were either adjusted or withdrawn also rose with CKD stage, this trend did not achieve statistical significance (See Table 4).

**Table 4:** Presents the distribution of patients, their medications, the requested modifications, and the changes that were accepted, stratified by CKD stage.

Stage (GFR)	Patients	Drugs	Patients		Drugs			
			No PIP n (%)	PIP n % p < 0.0001	Requests adjustment n (%) p = 0.0127	Adjusted n (%)	Requests withdrawal n (%) p < 0.0001	Withdrawn n (%)
1 (>90)	107	654	107 (100)					
2 (>60 AND <90)	348	2179	348 (100)					
3a (>45 AND <60)	126	888	91(72, 2)	35 (27,8)	45 (5,1)	13 (28,9)	7 (0,8)	2 (28,6)
3b (>30 AND <45)	74	652	33 (44,6)	41 (55,4)	54 (8,3)	16 (29,6)	14 (2,1)	8 (57,1)
4 (>15 AND < 30)	15	129	1 (6,7)	14 (93,3)	13 (10,1)	7 (53,8)	17 (13,2)	11 (64,7)
5 (>15)	0	0						
Total	670	4502	580 (86,6)	90 (13,4)	112 (2,5)	36 (32,1)	38 (0,8)	21 (55,3)

The medications for which changes were most frequently requested were olmesartan (27 times), metformin (26 times), and fenofibrate (12 times).

## Discussion

The primary objective of this study was not to conduct CKD screening; however, we found that 32.1% of patients had an eGFR below 60 ml/min/1.73 m^2^. This figure is considerably higher than the general CKD prevalence of 13.4% [4] but is quite similar to the 37.3% prevalence observed in individuals over 65 years of age [5,6], suggesting that our sample is fairly representative of the general population. Two studies carried out in Canadian community pharmacies [31,32] reported prevalences of 11.1% and 11.2%, respectively, among patients over 18 years of age. In contrast, a study conducted in the United Arab Emirates involving six pharmacies and 400 patients with CKD risk factors found a prevalence of 38.8%. In Spain, a screening study in community pharmacies focusing on high-risk populations is pending publication [33].

In a study very similar to ours [25], conducted in Spain with 22 pharmacies and a sample of 566 patients over 65 years old who were on at least three chronic medications, 266 patients were found to have an eGFR below 60 ml/min/1.73 m^2^, corresponding to 47.0% (95% CI: 42.9–51.1). When we restrict our sample to patients over 65 years old with at least three medications, the prevalence we obtain is 35.0% (95% CI: 30.9–39.1), which is slightly higher than our overall sample yet still markedly lower than the figure reported in the aforementioned study. These findings indicate that the prevalence of CKD is somewhat greater than 10% in the general population and approximately 40% in high-risk groups.

As anticipated, eGFR decreases with advancing age. In our study, the mean eGFR remained above 60 ml/min/1.73 m^2^ up to the age of 80, which is considered sufficient for dose adjustment purposes. However, in the 80–89 and ≥90 age groups, the mean eGFR fell below 60 ml/min/1.73 m^2^. A study involving 600 patients over the age of 85 reported an average eGFR of 42.1 ml/min/1.73 m^2^ [34].

The objective of this study was not to conduct CKD screening but to identify potentially inappropriate prescriptions (PIP) among patients with low eGFR. Among the 215 patients meeting this criterion, 90 (41.9%) had at least one PIP, which was subsequently reported to their primary care physician. This subgroup represents 13.4% of the total study sample. The identification of PIPs in 41.9% of patients with low eGFR supports the study’s justification and efficiency. Moreover, the proportion of patients requiring medication changes increased as eGFR decreased: 27.8% in CKD stage 3a, 55.4% in stage 3b, and 93.3% in stage 4. This trend is logical, as patients with poorer renal function are more likely to experience complications; however, one could also contend that these more severe patients receive greater medical attention, leading to prescriptions that already account for eGFR.

Polypharmacy also appears to influence the risk of potentially inappropriate prescriptions (PIP). Among patients with an eGFR < 60, those with PIP were taking an average of 2.3 more medications than those without PIP.

In comparison, Laville et al. [15] reported that 52% of a cohort of 3,033 CKD patients had at least one PIP, with 22% having two PIPs. A Scottish study [35] classified PIPs into three categories according to the British National Formulary 78 (2019): contraindicated, dose adjustment, and “potentially high risk – avoid if possible.” Their findings indicated that 3.9%, 15.2%, and 24.3% of patients had at least one PIP in each category, respectively, totaling 43.4%. Similarly, in Sweden, among a cohort of 30,372 patients over 65 years with stage 3 CKD and 2,161 patients with stage 4 CKD, inappropriate dosing was observed in 42.5% and 58.1% of cases, respectively, with contraindicated medications present in 9.4% and 38.0% [36]. In the United States [37], a cohort of 7,586 patients over 18 years old revealed that 4.9% had stage 3 or 4 CKD, of whom 46.6% had at least one PIP.

Another approach to evaluating potentially inappropriate prescriptions (PIPs) in CKD involves analyzing specific medications and determining the proportion of patients whose eGFR falls below the recommended level for a given drug dosage. Wood S [38] applied this method in 2018 across 80 practices in northern England by reviewing prescriptions for eight medications within a cohort of 70,900 patients over 65 years old. He found that 79.5% of patients over 85 using nitrofurantoin had an eGFR below the recommended threshold, whereas only 0.5% of NSAID users between 65 and 75 years old did so.

Furthermore, a systematic review of 49 studies [39] demonstrated a wide variability in the prevalence of PIPs in both hospital and outpatient settings, ranging from 9.4% to 81.1%. The review also highlighted that PIPs are associated with an increased risk of adverse effects, higher hospitalization rates, and greater mortality. Notably, in 21 of these studies, the effectiveness of interventions to correct PIPs was analyzed, with findings suggesting that such interventions are more successful when a clinical pharmacist directly communicates with the physicians.

All these studies have been conducted retrospectively through the review of clinical records, and there are comparatively fewer investigations performed by pharmacists. In Australia [40], a cohort of 365 nursing home residents was studied, revealing that 48% had CKD, and pharmacists identified at least one PIP in 16% of these patients.

In the context of community pharmacies involving direct patient interviews, only one study was found. Via-Sosa et al. [26] conducted a study in Spain across 18 community pharmacies, recruiting 440 patients, of whom 178 had low eGFR. These patients were prescribed a total of 1,092 medications, with 614 (56.2%) classified as PIPs. A total of 167 interventions were proposed, although only 31.4% of these were implemented.

Additionally, there is an ongoing randomized controlled trial in Australia involving 122 pharmacies, scheduled between March 2023 and May 2025, which combines CKD screening with medication analysis to determine the need for dose adjustments or the presence of nephrotoxic drugs [41].

Patient feedback on the pharmaceutical service for CKD risk assessment has been overwhelmingly positive, with 90% of patients reporting satisfaction and 88.9% agreeing to pharmacist referrals to their physicians [42].

### Strengths and limitations of the study

**Strengths:** This is a multicenter study conducted across different geographical settings, addressing a significant public health issue such as chronic kidney disease (CKD). The methodology is simple and easy to implement, utilizing a point-of-care (POC) device when necessary, and has been developed in collaboration with medical scientific societies. It holds potential for a meaningful impact on patient safety.

**Limitations:** The main limitation of this study is the absence of a control group, which hinders a definitive evaluation of the intervention’s actual effectiveness. Another significant limitation is the lack of communication with primary care physicians. We believe this issue substantially contributes to the low implementation rate of the proposed changes, which stands at 38%. This figure is comparable to the 31.4% reported by Via Sosa et al. [26], whose study—also conducted in Spain—encountered similar challenges related to interprofessional communication.

### Implications for research and practice

Although many PIPs are identified, this does not necessarily imply that they are truly inappropriate. It is essential for clinicians to assess the risk–benefit ratio in each case and tailor prescriptions to the individual patient’s situation. While community pharmacists can and should review patients’ medications and alert physicians to any detected PIPs, the final clinical decision rests with the physician.

The current method of communicating these findings to physicians—through reports delivered by patients during consultations—is suboptimal. There is a clear need to establish direct communication channels between community pharmacists and physicians, as such channels are currently lacking in Spain. Physicians are often unprepared to address the information contained in these reports, which they receive during patient encounters, limiting their ability to respond effectively. Direct communication between community pharmacists and physicians is highly desirable [43]. This need is supported by joint statements [44] from medical and pharmaceutical scientific societies, and several protocols [45] have already been developed to facilitate such collaboration.

The integration of community pharmacists into the multidisciplinary care team for renal patients is strongly recommended by the International Society of Nephrology [46].

## Conclusions

Spanish community pharmacies can play a crucial role in optimizing medication management for patients with reduced glomerular filtration rates by reviewing their prescriptions to identify potentially inappropriate prescriptions.
